# Effect of Pulsed Methylprednisolone on Pain, in Patients with HTLV-1-Associated Myelopathy

**DOI:** 10.1371/journal.pone.0152557

**Published:** 2016-04-14

**Authors:** Kevin G. Buell, Aiysha Puri, Maria Antonietta Demontis, Charlotte L. Short, Adine Adonis, Jana Haddow, Fabiola Martin, Divya Dhasmana, Graham P. Taylor

**Affiliations:** 1 Section of Virology, Department of Medicine, Imperial College London, Norfolk Place, London W2 1PG, United Kingdom; 2 National Centre for Human Retrovirology, Imperial College Healthcare NHS Trust, St Mary’s Hospital, Praed Street, London W2 1NY, United Kingdom; 3 Centre of Immunology and Infection, Hull York Medical School, Department of Biology, University of York, Heslington, York YO10 5DD, United Kingdom; George Washington University, UNITED STATES

## Abstract

HTLV-1-associated myelopathy/tropical spastic paraparesis (HAM/TSP) is an immune mediated myelopathy caused by the human T-lymphotropic virus type 1 (HTLV-1). The efficacy of treatments used for patients with HAM/TSP is uncertain. The aim of this study is to document the efficacy of pulsed methylprednisolone in patients with HAM/TSP. Data from an open cohort of 26 patients with HAM/TSP was retrospectively analysed. 1g IV methylprednisolone was infused on three consecutive days. The outcomes were pain, gait, urinary frequency and nocturia, a range of inflammatory markers and HTLV-1 proviral load. Treatment was well tolerated in all but one patient. Significant improvements in pain were: observed immediately, unrelated to duration of disease and maintained for three months. Improvement in gait was only seen on Day 3 of treatment. Baseline cytokine concentrations did not correlate to baseline pain or gait impairment but a decrease in tumour necrosis factor-alpha (TNF-α) concentration after pulsed methylprednisolone was associated with improvements in both. Until compared with placebo, treatment with pulsed methylprednisolone should be offered to patients with HAM/TSP for the treatment of pain present despite regular analgesia.

## Introduction

The human T lymphotropic virus type 1 (HTLV-1) was recognised as the first human retrovirus, with oncogenic potential, following its discovery by independent groups in the USA[1] and Japan[2]. The prevalence of HTLV-1, estimated at 5–10 million worldwide, is variable between and even within countries[3]. High prevalence, defined as greater than 1:10,000 of the general population or greater than 1:100 first time blood donors, is reported in Japan, the Caribbean, Western and Southern Africa, South America and Melanesia[4]. European countries have a low prevalence with the exception of Romania[4]. Gessain *et al* first reported the high frequency of HTLV-1 antibodies in patients with tropical spastic paraparesis (TSP) in Martinique in 1985[5] whilst Osame *et al*. coined the term ‘HTLV associated myelopathy’ (HAM) following the identification of HTLV-1 antibodies in a cohort of patients with a similar myelopathy in Japan in 1986[6].

A minority of HTLV-1 infected persons develop symptoms and estimates of risk of HAM/TSP among HTLV-1 carriers vary from estimated life time risks of 0.25% in Japan[7] and 1.9% in Jamaica and Trinidad[8] to an observed prevalence of 2.4% among US blood donors[9] and a 1.5% incidence during up to 8 years observation in cohort in Brazil[10]. A high HTLV-1 proviral load in carriers increases the likelihood of HAM/TSP in comparison to those with lower proviral with the probability of disease increasing exponentially above the threshold of 1% HTLV-1 infected peripheral blood mononuclear cells (PBMC)[11].

HAM/TSP is characterised by perivascular lymphocytic infiltrates in the brain and spinal cord, initially CD4+ lymphocytes[12], followed by predominantly CD8+ lymphocytes[13] and later by atrophy. These findings, along with evidence of HTLV-1 specific CD4+[14,15] and CD8+ [16–18] in the circulation (and CD8+ cells in the cerebrospinal fluid[19]) have led to the now favoured bystander theory of pathogenesis in which in response to HTLV-1 infection within the central nervous system activated cytotoxic T lymphocytes (CTLs) produce cytokines that not only recruit additional inflammatory cells but are neurotoxic, resulting in the development of chronic inflammatory lesions and demyelination in the spinal cord[20].

The most common clinical findings in HAM/TSP are lower limb weakness and hyperreflexia[21]. Lower limb weakness progressively affects gait and patients’ needs change from walking unaided, to utilising one or two crutches and finally, around one in five patients with symptoms for more than ten years become wheelchair-dependent[22–25]. 60–88% of patients with HAM/TSP report pain [26–28] and this is associated with decreased functional capacity and lower quality of life[29].

Nearly 35 years after the discovery of HTLV-1 there is still no standardized treatment for HAM/TSP. The evidence underlying current therapy principally originates from case reports or small cohorts and only two randomised controlled studies have been completed[30,31]. The list of treatments tried is extensive and includes interferon alpha (IFN-Ɣ)[30], antiretroviral therapies[31], immunosuppression[32], anabolic steroids[33] and Vitamin C[34]. Araujo *et al* reported sustained benefit following high dose methyl prednisolone in one patient (out of 23), with recent onset symptoms[35]. In Kagoshima three of 10 patients with relatively rapid progression of motor dysfunction showed excellent to moderate responses treated with pulsed IV methylprednisolone[36].

Pulsed methyl prednisolone has been used in a variety of immune-mediated diseases. A clinical trial in children with lupus nephritis concluded that the benefits of pulsed methylprednisolone were equivalent to high dose oral prednisolone but with fewer adverse effects[37]. In a double-blind randomised controlled trial of 2.5g methylprednisolone administered in equal doses over 5 days IV methyl prednisolone outperformed placebo in patients with multiple sclerosis particularly during acute relapse but with some benefit during chronic progression[38]. Although toxicity has been reported no increase in adverse medical events including avascular necrosis of the hip was observed in comparison with control group of patients with rheumatoid arthritis when a retrospective double-blind review was conducted[39].

Based on the limited reports of steroid use in patients with HAM, the anti-inflammatory effects reported in other conditions and an established safety profile, pulsed methyl prednisolone has been offered to patients with HAM/TSP attending the National Centre for Human Retrovirology. To test the hypothesis that one course of pulsed IV methylprednisolone is beneficial in patients with HAM a retrospective analysis of data from treated patients was conducted. Key symptoms, signs, HTLV-1 proviral load and markers of inflammation were used to measure the effect of pulsed IV methylprednisolone in a well characterised population of patients with HAM/TSP.

## Materials and Methods

Patients attending the National Centre for Human Retrovirology (NCHR) at St. Mary’s Hospital, London are invited to participate in HTLV research through the Communicable Disease Research Biobank (Oxford REC C Committee Ref: 09/H0606/106). After giving written informed consent, allowing clinical data analysis, patients donate blood for research purposes, which is stored under The Human Tissue Act licence on the St. Mary’s Campus.

Since its foundation in 1991, 111 patients have attended the centre for management of HAM/TSP. Pulsed IV methylprednisolone has been offered as a treatment since 2004 with 28 patients treated up to May 2014. Notes from two patients could not be retrieved. The diagnosis of HAM/TSP in the remaining 26 was in accordance with WHO criteria[40] with characteristic, predominantly motor signs in the lower limbs: weakness and/or spasticity, hyper-reflexia with Babinksi’s sign; detection of anti-HTLV-1 antibodies, and exclusion of other potential causes of a spastic paraparesis. Twenty-five patients received the standard 1g methylprednisolone on three consecutive days: D0, D1, and D2; and one patient received one 500mg IV methylprednisolone dose only. The clinical parameters of the cohort at D0 are summarised in Table 1. At the time of treatment their median age was 54 years (range 20–80 years) and the median duration of HAM/TSP was 8.3 years. Seventeen patients used a crutch or walker and four patients were wheelchair-bound.

**Table 1:** Baseline demographics of the patients treated with methylprednisolone.

	Male n = 7	Female n = 19	All n = 26
Median age(range) (in years)	62.7(47.1–73.2)	52.6(19.9–79.9)	53.7(19.9–79.9)
Median duration of disease(range) (in years)	5.8(0.7–19.4)	9.8 (0.9–28.1)	8.3(0.7–28.1)
Afro Caribbean (%)	4 (57)	11 (57.9)	15 (57.7)
African (%)	1 (14)	4 (21)	5 (19.2)
Caucasian (%)	2 (29)	2 (10.5)	4 (15.4)
Other (%)	0 (0)	2 (10.5)	2 (7.7)
Patients on otherimmunosuppressant therapy (%)	0 (0)	2* (10.5)	2 (7.7)
Patients wheel chair bound at D0 (%)	1 (14.3)	3 (15.8)	4 (15.4)
Patients using crutches or walker at D0 (%)	4 (57.1)	13 (68.4)	17 (65.4)
Patients with suprapubic catheters at D0 (%)	0 (0)	2 (10.5)	2 (7.7)
Patients using intermittent self-catheterisation at D0 (%)	0 (0)	5 (26.3)	5 (19.2)

* One patient started Hydroxychloroquine 200mg bd 18 weeks after IV methylprednisolone and one patient was on Methotrexate 12.5mg weekly throughout the period of observation.

To ascertain whether patients had stable, improving or worsening symptoms of HAM/TSP in the period leading up to treatment, data from the appointment immediately preceding D0 (Pre) were also collected. The duration of any effect attributed to treatment was determined from three follow up (FU) appointments: FU1 at week 4 ± 2 weeks, FU2 at week 12 ± 5 weeks and FU3 at week 24 ± 6. As not all patients were included for all parameters studied or at every time point the mean number of weeks between D0 and FU1-3 varied marginally for each parameter.

The clinical parameters assessed were pain, gait, urinary frequency and nocturia. Lower back/limb pain was measured using a self-reported visual analogue 11 point scale (VAS) with 0 representing no pain and 10 the most severe pain that the respondent could imagine. Gait was assessed by a standardised 10 meter timed walk (10m TW) measurement whereby the time taken by the patient to walk 10 meters on the level, with the assistance of their usual walking aid, from a standing position, was recorded in seconds. Patients were asked to recall the average number of micturition and/or intermittent self-catheterisation (ISC) episodes per day (frequency) and after retiring to sleep (nocturia) since the previous assessment. A change in the use of walking aid and catheterisation was not included in the analysis of continuous data but was instead incorporated into the analysis of categorical data. A change in walking aid or catheterisation was considered to represent a larger clinical change than any contradictory increase or decrease in 10m TW, frequency or nocturia. The following laboratory parameters were routinely documented and measured by a clinical pathology accredited laboratory according to standard methodology: alanine aminotransferase (ALT), creatine kinase (CK), haemoglobin (Hb), lactate dehydrogenase (LDH), white blood cell count. HTLV-1 proviral loads (pVL) were quantified using a real-time polymerase chain reaction and expressed as a percentage of PBMC infected[41]. The prevalence of urinary tract infections (UTIs) was determined by a point of care test followed, where indicated, by urine culture.

A cytokine profile was conducted in a subset of nine patients with suitable, undiluted, peripheral blood plasma samples available from D0, D2 and a single follow-up appointment, a mean of 10 ± 4.3 weeks from D0. IFN- γ, IL-10, IL-12 p70, IL-13, IL-1B, IL-2, IL-4, IL-6, IL-8 and TNF-α were measured using the Meso Scale Discovery Multi Spot Assay System proinflammatory panel 1 assay, according to the instructions provided by the manufacturer (Meso Scale Diagnostics, Rockville USA). All assays were duplicated and the mean was taken for the cytokine concentration at each time point.

Continuous parameters were analysed using paired T tests to assess whether the difference between Pre, D2, and FU1-3 compared to D0 was statistically significant (p<0.05). The data from Pre, D2, and FU1-3 were categorised into small (0.1–30%) or large (>30%) improvements and deteriorations in comparison to D0. Chi squared goodness of fit tests were used to assess categorical change after treatment at D2, and FU1-3 with the pre-treatment change from Pre to D0. Linear correlation between two parameters was assessed using Pearson product-moment correlation coefficient of the sample, ‘r’. Correlation was considered strong if r>0.5, weak if 0.20<r<0.49, and minimal if r<0.19. A table of critical values for Pearson’s correlation using p<0.05 was used to determine whether the correlation was statistically significant.

## Results

Of the 25 patients treated with 1g IV Methylprednisolone on three consecutive days, 24 completed the treatment without any side effects. One patient became hypomanic and discontinued treatment after the first dose. The patient originally prescribed the single 500mg dose tolerated the treatment well.

### Pain

**Fig 1:**
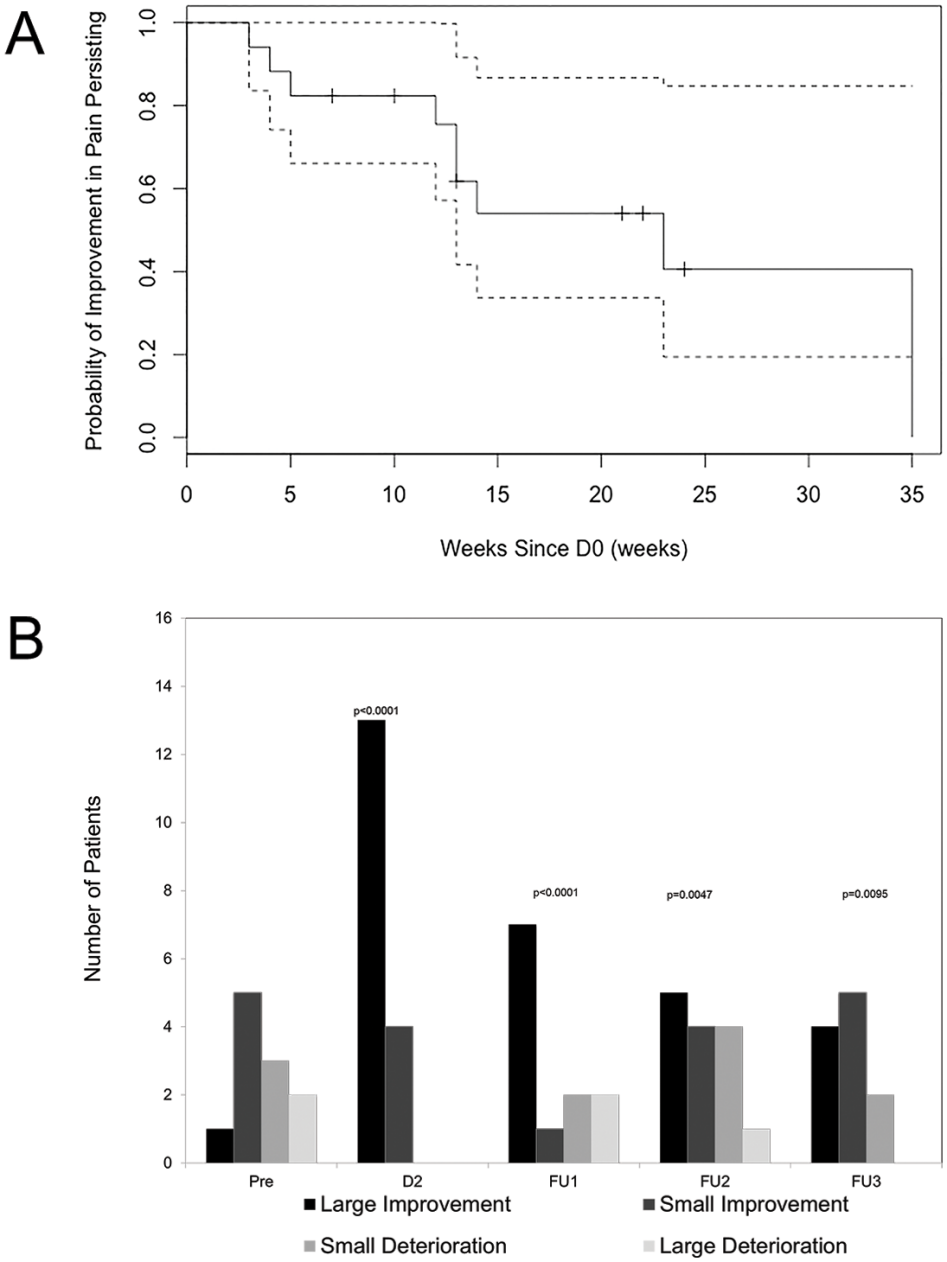
Effect of methyl prednisolone on pain. A, Survival curve showing the duration of improvement in pain. Dotted lines represent confidence intervals. B, Change in pain as measured by the visual analogue scale. Bar chart representation of the number of patients with a large improvement (>30%), small improvement (30%-0.1%), small deterioration (0% - 30%), and large deterioration (>30%) in comparison to D0. P values are goodness of fit chi square tests assessing the categorical change after treatment at D2, and FU1-3 with the pre-treatment change from Pre-D0.

Eighteen patients (16 female and two male) had documented pain at baseline and were thus included in the pain analysis. Eight patients were excluded including seven that were pain-free at D0 and one patient whose relevant volume of medical records could not be retrieved. Pain scores improved at D2 in 17/18 patients with a mean maximum improvement of 3.8 points at D2 (p<0.0001) (Table 2). The pain score of patient 18 was not recorded at D2. The prevalence of the improvement in pain gradually decreased over time. As shown in Fig 1A 75% still reported benefit 12 weeks from D0 during which time only one subject had a change in analgesia (see below). Although the improvement in pain remained statistically significant at FU3, six months after treatment (Table 2) only 40% of subjects reported improvement at this point and 10/18 had made changes to analgesia. The reduction in pain was not statistically significant beyond this point; evaluated at 9 and 12 months from D0 respectively (data not shown). To describe the variation in degree of improvement at the individual level pain was arbitrarily categorised into small (0.1–30%) or large (>30%) improvements or deteriorations in comparison to D0. As shown in Fig 1B all 17 patients with documented pain at D0 improved at D2, with 13 (76%) patients improving by more than 30% (p<0.0001). A higher (worse) baseline pain score at D0 correlated weakly to a larger % improvement in pain from D0 to D2 (data not shown). The duration of disease preceding treatment did not correlate with the degree of improvement in pain from D0 to D2 (data not shown).

**Table 2:** Summary of clinical parameters at each visit.

CD8% Viral Load	Pre	D0	D2	FU1	FU2	FU3
Pain						
Median (IQR)	*8 (5–8*.*5)*	*7 (6–8)*	***3 (1*.*5–5)***	***5 (2–7)***	***6*.*5 (4*.*3–8)***	***5 (3*.*5–6)***
Mean (SD)	*6*.*6 (3*.*2)*	*7*.*1 (1*.*8)*	***3*.*3 (2*.*6)***	***4*.*5 (2*.*9)***	***5*.*6 (3*.*4)***	***5*.*0 (2*.*3)***
P value	*0*.*9*	*NA*	***<0*.*0001***	***0*.*03***	***0*.*04***	***0*.*005***
10mTW (secs)						
Median (IQR)	*25*.*8 (16*.*2–34*.*9)*	*23*.*7 (17*.*0–32*.*0)*	***22*.*3 (14*.*6–27*.*7)***	*20*.*8 (17*.*0–26*.*7)*	*23*.*1 (18*.*1–25*.*9)*	*23*.*7 (17*.*2–25*.*4)*
Mean (SD)	*28*.*9 (18*.*9)*	*32*.*0 (27*.*1)*	***24*.*0 (13*.*4)***	*30*.*3 (25*.*6)*	*26*.*2 (16*.*0)*	*25*.*6 (19*.*5)*
P value	*0*.*2*	*NA*	***0*.*03***	*0*.*3*	*0*.*7*	*0*.*7*
CD4%						
Median (IQR)	*43 (36–56)*	*48 (41–57)*	***36 (28–39)***	*47 (39–55)*	*48 (36–52)*	*42 (30–49)*
Mean (SD)	44 (13)	47 (12)	**34 (9)**	46 (14)	44 (15)	40 (14)
P value	0.3	NA	**<0.0001**	0.9	0.5	0.5
CD8%						
Median (IQR)	*27 (22–39)*	*24 (18–31)*	***20 (16–25)***	*24 (20–33)*	*25 (22–36)*	*32 (25–51)*
Mean (SD)	33 (19)	26 (12)	**22 (9)**	31 (19)	32 (18)	37 (21)
P value	0.04	NA	**0.02**	0.4	0.4	0.9
HTLV proviral load						
Median (IQR)	*15*.*5 (8*.*6–25*.*7)*	*10*.*9 (4*.*3–21*.*3)*	*3*.*9 (1*.*1–22*.*2)*	*11*.*1 (4*.*6–23*.*8)*	*11*.*5 (5*.*7–19*.*7)*	*7*.*2 (2*.*3–13*.*3)*
Mean (SD)	18.5 (12.7)	15.9 (17.5)	11.1 (13.4)	16.5 (17.1)	14.7 (14.1)	9.0 (7.3)
P value	0.3	NA	0.09	0.2	0.5	0.1

Where there is a statistically significant differences from D0 (p<0.05) this is highlighted in bold font.

All the patients with pain were on analgesia at time of infusions and during follow up. To determine whether the observed improvement in pain might be attributable to changes in analgesia rather than associated with the methylprednisolone infusions analgesia at each visit was reviewed. During follow up, ten patients increased their use of analgesia and in five cases this was followed by an improvement in pain score. However, with the one exception, analgesia was modified at or after FU2 (12 weeks) i.e. after the effect of methyl prednisolone had been documented.

### Ambulation

**Fig 2:**
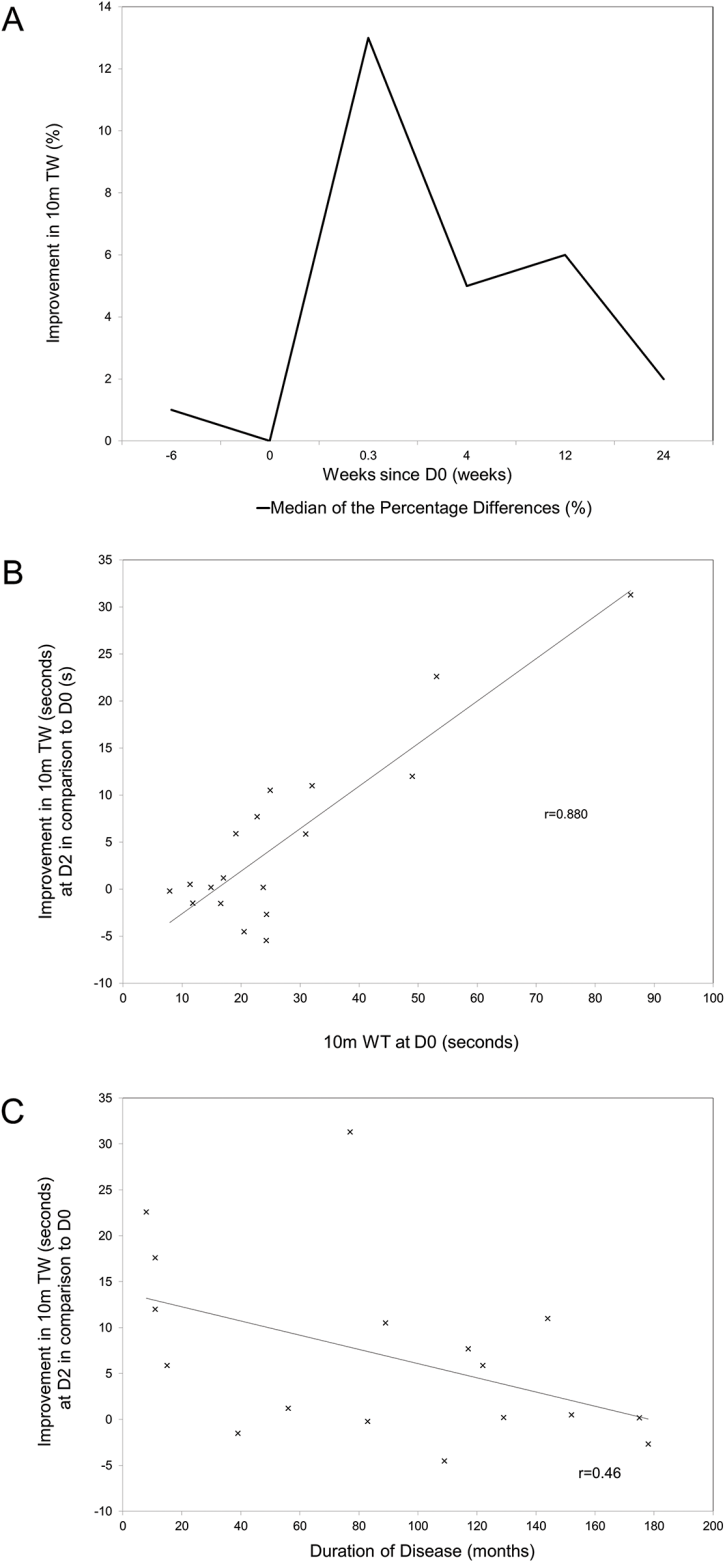
Effect of methyl prednisolone on gait. A, Change in 10m timed walk as the median of the percentage difference (%) in comparison to D0. B, Correlation between the 10m TW (seconds) at D0 and an improvement in the 10m TW from D0 to D2 (seconds) using Pearson’s correlation coefficient of the sample (r). Outliers were excluded. C, Correlation between duration of disease and improvement in 10m TW. Scatter plot with line of best fit showing duration of disease (months) against the improvement in the 10m TW (seconds) at D2 using Pearson’s correlation coefficient of the sample (r). Outliers were excluded.

Twenty-two ambulant patients were included in the 10m TW analyses. The improvement in 10 TW was maximal at D2 with a mean 8.0 seconds per 10m improvement (s/10m) (p = 0.024) (Table 2) but this was not sustained to FU1. As shown in Fig 2A, this maximal effect equates to a median 13% improvement in the time taken to walk 10m. Only the improvement in 10m TW at D2 was statistically significant (Table 2). As shown in Fig 2B, the magnitude of improvement in 10m TW correlated strongly with a slower baseline 10m walk (r = 0.88). The improvement in 10m TW at D2 also correlated with a shorter duration of disease, r = 0.46 (Fig 2C). All four patients treated within 24 months of their first symptom experienced an improvement in 10m TW. Four patients use of walking aid increased during the 24 weeks of observation.

### Urinary symptoms

No significant changes in urinary frequency or nocturia were observed, these remaining constant at four and two episodes respectively. Four patients, of whom two had SPCs, had documented UTIs during three day infusions and two additional patients, not using catheters, reported UTIs at FU1. All of which were adequately treated without further complications and there was no report of UTIs beyond FU1.

### Biochemical

Fluctuations in creatine kinase, alanine transaminase, lactate dehydrogenase and haemoglobin were observed but with no consistent pattern and these were not statistically significant (data not shown). Temporary neutrophilia and lymphocytopaenia at D2 were statistically significant (p<0.0001) with a greater decrease in CD4+ T-cells than CD8+ T-cells as shown in Table 2. Following the same trend, median HTLV-1 proviral load decreased transiently from 10.9% at D0 to 3.9% at D2 but this did not reach statistical significance (p = 0.086) and had returned to baseline by FU1. The percentage change in CD4% from D0 to D2 did not correlate with the percentage change in HTLV-1 proviral load from D0 to D2 (data not shown).

### Cytokines

The median concentration of each of the eight pro-inflammatory cytokines (IFN-y, IL-12 p70, IL-1B, IL-2, IL-4, IL-6, IL-8 and TNF-α) decreased from D0 to D2 and the anti-inflammatory cytokine IL-10 increased. Only the reductions in IFN-γ (p = 0.003), IL-6 (p = 0.044) and TNF-α (p = 0.004) were statistically significant at D2 (Table 3). The decrease in TNF-α alone remained statistically significant 10 weeks from D0 at FU (p = 0.032).

**Table 3:** Summary of cytokine concentration in response to pulsed IV methylprednisolone.

	D0	D2	FU
INF-y (pg/ml)	1.3	0.005**	0.5
IL-6 (pg/ml)	0.01	0.001*	0.008
TNF-α (pg/ml)	0.3	0.06**	0.2*

Median concentrations (pg/ml) of TNF-α, IL-6, and IFN-Ɣ at D0, D2 and FU are shown, where statistically significant differences from D0 are indicated by one asterisk if p < 0.05 and two asterisks if p < 0.01.

**Fig 3:**
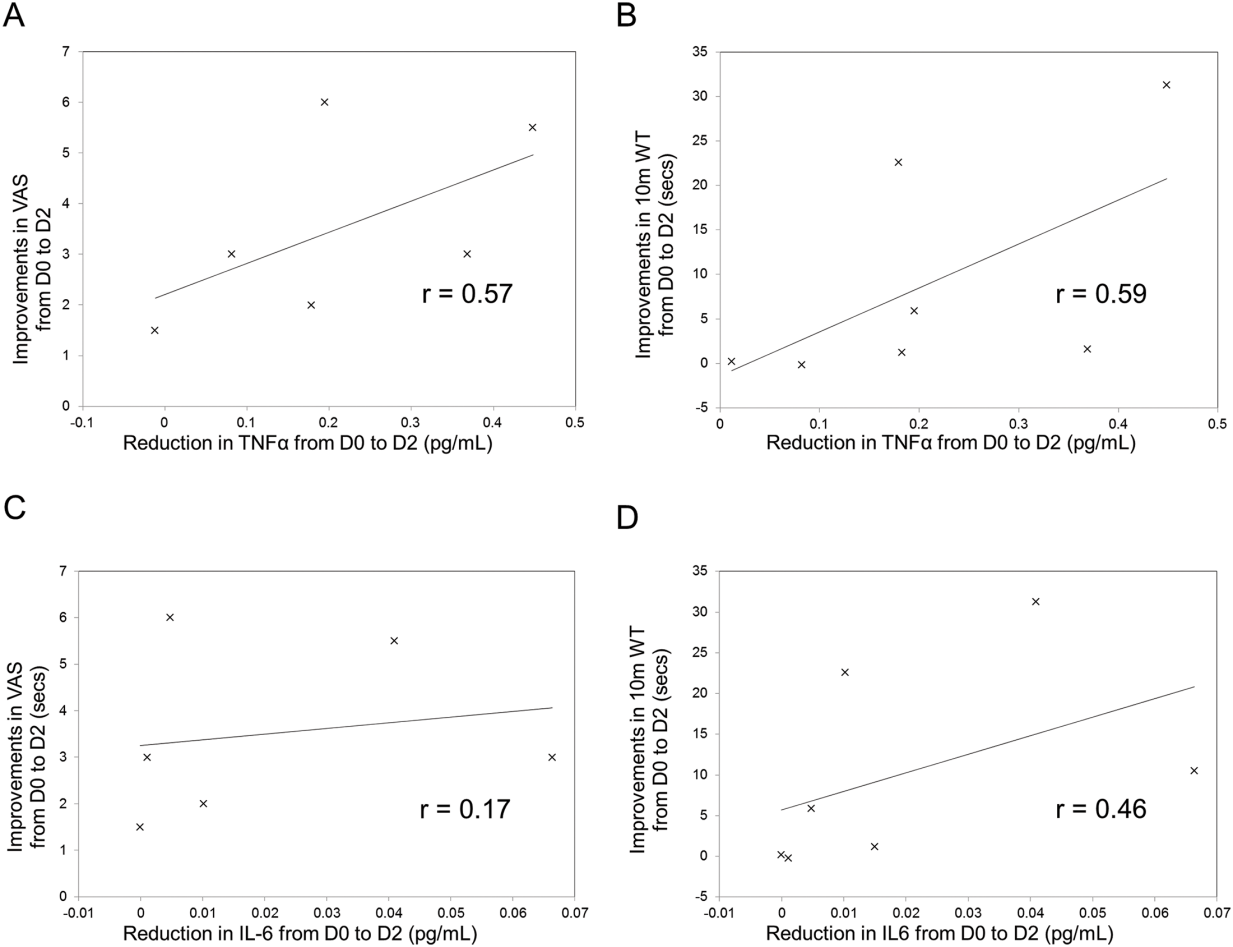
Correlations between cytokines, pain and gait. Pearson correlation coefficient of the sample (r) was used to assess the correlation between reductions in TNF-α and IL-6 concentration (pg/ml) with improvements in the 10m TW (seconds) and pain (VAS) from D0 to D2. **A)** Correlation between TNF-α concentration and pain. **B**) Correlation between TNF-α concentration and 10m TW. **C**) Correlation between IL-6 concentration and pain. **D**) Correlation between IL-6 concentration and 10m TW.

Pain at baseline correlated moderately with IL-6 (r = 0.36), weakly with TNF-α (r = 0.13), and not with IFN-Ɣ (r = -0.09) concentrations at D0 (data not shown). The 10m TW at D0 correlated moderately to IFN-Ɣ at D0 (r = -0.30), weakly with TNF-α (r = 0.24), and minimally with IL-6 (r = 0.16) concentrations at D0 (data not shown). As shown in Fig 3, the improvement at D2 in pain (r = 0.57) and 10m TW (r = 0.59) correlated strongly with the reduction in TNF-α concentration. The change in IL-6 concentration correlated moderately with the improvement in 10m TW (r = 0.46) and minimally to the reduction in pain (r = 0.17). Changes in IFN-Ɣ concentrations correlated poorly with changes in pain and gait.

## Discussion

Pain is a common symptom of HAM/TSP, reported in up to 88% of patients[28]. The effect of pulsed methyl prednisolone on pain in patients with HAM/TSP has not previously been reported. In our cohort, 18 (67%) patients reported pain at baseline. It is important to note that the improvement in pain following pulsed methylprednisolone was achieved in patients that remained in pain despite many prior and current treatments for pain. All patients reported an improvement, mostly commonly a greater than 30% improvement, in pain score following three doses of pulsed IV methylprednisolone. The effect was seen immediately and was maximal by the time of the third dose. As a cohort, the benefit thereafter decreased steadily but remained significant for 3 months after this pulsed therapy. Although the effect was not maintained in all patients, approximately one in three patients still reported improvement after six months although changes in analgesia make firm conclusions after 12 weeks difficult. Although steroid treatments are known to cause a transient effect, the benefit from retreating patients with repeated cycles of pulsed IV methylprednisolone could provide long-term pain control in HAM/TSP patients.

It is unlikely that a change in other medication was responsible for the statistically significant reduction in pain observed prior to 12 weeks. Of the 10 patients who reported increased use of analgesia during follow up, only five reported an improvement in pain and in four of these patients the medication was altered at or after FU2, 12 weeks after treatment, by which time the majority of the pain effect observed with methylprednisolone had waned. The immediate effect of pulsed IV methylprednisolone was not influenced by a change in analgesia however the prolonged improvement in pain seen in a subset may in part be attributed to the addition of other analgesia.

The effect of pulsed IV methylprednisolone on gait was less striking. The improvement in walking time for 10m was only statistically significant on the last day of treatment and not thereafter. However, the magnitude of the effect on gait was predicted by both the duration of disease and the baseline 10m TW. Patients with shorter duration of disease and more severe gait impairment had the greatest improvement in gait. Araujo *et al*. speculated that patients with a shorter history would benefit more from treatment[35]. In the present cohort this was the case for gait but not for pain where the duration of symptoms did not predict the magnitude of the analgesic effect.

Changes in plasma cytokines in relation to pulsed IV methylprednisolone have been reported in other diseases such as Stevens Johnson Syndrome[42] but they have not been assessed previously in HAM/TSP. Cytokine profiles were analysed before, during and after pulsed IV methylprednisolone. The concentrations of all pro inflammatory cytokines decreased with methylprednisolone, in parallel with the improvements in pain and gait at D2. Only the changes in TNF-α, IL-6 and IFN-Ɣ reached statistical significance however this may be due to the small size of this subset. Whether there is a causal link between TNF-α and the low back and lower limb pain in patients with HAM/TSP is uncertain, with absolute baseline levels not predicting the severity of the pain, whereas a reduction in TNF-α correlated with improvement in pain. Additional factors including inter-patient differences in the threshold for TNF-α concentration to be associated with pain, may contribute to this discrepancy but unless the findings are entirely coincidental the data point to the role of inflammation in the pain experienced by patients with HAM/TSP.

Baseline cytokine values did not predict more severe gait deficit but reductions in TNF-α, and IL-6 were linked to an improvement in gait. One interpretation of these observations is that the neurological deficit that is associated with impaired gait is either more fixed or less steroid responsive than the inflammation that is associated with pain. These observations, suggest that more targeted therapies might be selected for the treatment of HAM/TSP. Whilst anti-TNF therapies would be a natural choice based on these findings limited experience with infliximab monotherapy was tempered by a high rate of adverse events and cessation of the study for futility[43]. Targeted IL-6 inhibition has not been reported in patients with HAM.

Steroid sparing agents can also be considered—in an open pilot study ciclosporin was associated with sustained improvement in pain and mobility[32].

Since glucocorticoids are immunosuppressant and HTLV-1 pVL is associated with the risk of both HTLV-1-associated inflammatory disease and ATLL, HTLV-1 pVL was monitored during, and for six months after, the high dose IV methylprednisolone therapy. A transient decrease in pVL was observed during the three days of the infusions, which was initially attributed to glucocorticoid induced redistribution of the intravascular lymphocyte pool to the bone marrow[44–46]. However, the reduction in circulating CD4 T-lymphocytes did not account for the reduction in HTLV-1 pVL with poor correlation between these two measures at the individual level. A selective effect of methylprednisolone on HTLV-1 infected T-cells remains a possibility with either selective sequestration or apoptosis potential explanations. However, pVL returned to the baseline by week 4. Perhaps more importantly no increase in pVL was observed during six months follow-up which suggests that, at least in this regard, methylprednisolone can be administered safely to patients with HAM/TSP without loss of immune control of the viral load.

UTIs are common in patients with HAM/TSP especially those who practice intermittent self-catheterisation[47]. Although six patients developed UTIs, these occurred early, were detected and treated promptly without any sequelae.

The literature underlying the use of IV methylprednisolone in HAM/TSP is limited and to our knowledge has only been described in case reports[48–50] and three observational studies[35,36,51]. This is the second largest study of pulsed methylprednisolone in HAM/TSP and is the first to focus on the effectiveness of pulsed IV methylprednisolone in the management of pain in patients with HTLV-1 associated myelopathy. In addition, we characterise changes in pVL and cytokine concentrations during treatment and demonstrate correlation between the duration of disease and treatment response. Although the improvement was transient HAM/TSP is a progressive disease with a mean deterioration in 10m TW of 4 seconds per annum in this cohort[23]. That 10m TW remained non-statistically improved compared with baseline indicates that the possibility that pulsed methyl prednisolone modified this progression cannot be excluded.

These data support the use of pulsed IV methylprednisolone in patients whose motor abilities deteriorate rapidly as reported by Nakagawa *et al*[36] who used pulsed IV methylprednisolone solely with the aim of improving gait, as well as for patients with pain despite analgesia. However, this study reports on a single course of methylprednisolone and therefore benefits from repeated cycles cannot be assumed. Croda *et al*. treated their patients every 3–4 months, for an average of 3.4 cycles, with most improvement observed following the first two cycles and decreasing benefit thereafter [51]. Importantly Croda *et al* did not measure pain outcomes.

This retrospective study has a number of weaknesses including potential for placebo effect, potential for selection bias and reliance on data collection at routine clinic visits. However, methyl prednisolone is widely accessible and the data indicate an effect which should now be tested in a randomised control trial to accurately assess its use in patients with HAM/TSP.

### Summary

A three-day course of 1g IV methylprednisolone was associated with a significant improvement in pain in patients with HAM/TSP lasting at least three months with benefit independent of duration of HAM/TSP. The effect of methyl prednisolone on gait was limited with no benefit apparent four weeks after therapy. The clinical benefits coincided with reductions in plasma concentration of pro-inflammatory cytokines TNF-α and IL-6 Pulsed steroid therapy was not associated with any long term effect on HTLV-1 proviral load.
